# “The patient is awake and we need to stay calm”: reconsidering indirect communication in the face of medical error and professionalism lapses

**DOI:** 10.1186/s41077-024-00293-4

**Published:** 2024-05-10

**Authors:** Taryn Taylor, Lauren Columbus, Harrison Banner, Natashia Seemann, Trevor Hines Duncliffe, Rachael Pack

**Affiliations:** 1 Department of Obstetrics & Gynaecology, London Health Sciences Centre, Victoria Hospital, 800 Commissioners Rd E, London, ON N6A 5W9 Canada; 2 Centre for Education Research & Innovation, Schulich School of Medicine and Dentistry, Western University, London, Canada; 3 Department of Midwifery, London Health Sciences Centre, London, Canada; 4 Department of Surgery, London Health Sciences Centre, London, Canada; 5 Faculty of Health & Rehabilitation Sciences, Western University, London, Canada

**Keywords:** Simulation primed inquiry, Qualitative research, Speaking-up, Communication, Teamwork

## Abstract

**Background:**

Although speaking up is lauded as a critical patient safety strategy, it remains exceptionally challenging for team members to enact. Existing efforts to address the problem of silence among interprofessional teams involve training low-authority members to use direct language and unambiguous challenge scripts. The role or value of indirect communication in preventing medical error remains largely unexplored despite its pervasiveness among interprofessional teams. This study explores the role of indirect challenges in the face of medical error and professionalism lapses.

**Methods:**

Obstetricians at one academic center participated in an interprofessional simulation as a partial actor. Thirteen iterations were completed with 39 participants (13 obstetrician consultants, 11 obstetric residents, 2 family medicine consultants, 5 midwives, and 8 obstetrical nurses). Thirty participants completed a subsequent semi-structured interview. Five challenge moments were scripted for the obstetrician involving deliberate clinical judgment errors or professionalism infractions. Other participants were unaware of the obstetrician’s partial actor role. Scenarios were videotaped; debriefs and interviews were audio-recorded and transcribed verbatim and analyzed using a constructivist qualitative approach.

**Results:**

Low-authority team members primarily relied on indirect challenge scripts to promote patient safety during simulation. Faculty participants were highly receptive to indirect challenges from low-authority team members, particularly in front of awake patients. In the context of obstetric care, direct challenges were actually viewed by participants as threatening to patient trust and disruptive to the interprofessional team. Instead of exclusively focusing our efforts on encouraging low-authority team members to speak up through direct challenges, it may be fruitful to expand our attention toward teaching faculty to identify, listen for, and respond to the indirect, subtle challenges that are already prolific among interprofessional teams.

**Supplementary Information:**

The online version contains supplementary material available at 10.1186/s41077-024-00293-4.

## Background

Efforts to improve direct communication within interprofessional healthcare teams have largely focused on training low-authority team members to be brave and speak up explicitly despite great interpersonal risk [1–4]. Existing research focuses on encouraging team members to use challenge scripts or heuristics intended to ease the burden of speaking up [1]. These interventions have been largely unsuccessful [4], in part because they overlook the role and responsibility of the team leader in establishing a work environment in which it is safe to challenge authority. Barriers to using direct communication when speaking up within a healthcare context are well-established, including fear of being wrong, fear of retribution, and fear of conflict [5, 6]. These are not insignificant barriers. Research has shown that these fears often preclude team members from speaking up even if doing so could prevent certain, significant patient harm [5, 7]. Thus, it is unsurprising that educational interventions aimed at low-authority team members simply practicing specific scripts may not solve the problem of silence within interprofessional teams.

Furthermore, these efforts assume that direct or explicit communication is always superior and preferable to indirect communication, particularly during acute scenarios when the team leader is faltering [2]. The Elaine Bromley case represents one of the most evocative examples often cited to emphasize the moral imperative to use direct communication in such situations [1]. Ms. Bromley died tragically because the perioperative team was unexpectedly unable to intubate or ventilate her and the anesthetist did not establish a surgical airway, which would likely have saved her life [8]. An independent inquiry into her death revealed that multiple team members were acutely aware of the urgent need for surgical intervention, but their low authority status prevented them from speaking up directly [8].

The emphasis on direct expressions of concern and the use of specific challenge scripts arises from the aviation safety literature [3]. However, unlike the cockpit where aviation teams can communicate candidly beyond their passengers’ listening ears, some healthcare teams (for example, in obstetrics) must communicate in front of their awake patients, often in acute, high-stakes scenarios. This may partly explain why most of the existing healthcare speaking-up literature is situated in perioperative or critical care teams, with patients who are often fully anesthetized and thus not typically part of the speaking-up equation [2, 3, 7, 9, 10].

The resulting over-emphasis on the importance of directly challenging team leaders has led to a troubling under-exploration of the potential role of indirect communication within interprofessional teams. Given that there are so many barriers to direct communication, we set out to explore the potential role of indirect challenges in interprofessional teams when faced with medical errors and professionalism lapses enacted by the team leader.

## Methods

We used a simulation-primed elicitation approach to this constructivist qualitative study [11] as it is advantageous for exploring sensitive topics. This approach enabled us to have rich discussions during the group debriefs and individual follow-up interviews, informed by participants’ behaviors and thought processes during the simulation scenario, rather than relying exclusively on participant recall from other similar real-life situations.

### Recruitment and participants

Participants were recruited via email and word of mouth from a tertiary academic hospital in Ontario, Canada. All OB consultants, OB residents, family medicine consultants, midwives, and obstetrical nurses were invited to participate in the study. In the initial phases of recruitment, all interested practitioners from the above groups were enrolled in the study. As data collection continued, we used purposive sampling techniques to target specific groups (i.e., family medicine consultants) to ensure that our final sample reflected the diversity of teams that routinely work together in our practice setting. Study participants are detailed in Table 1. Study participation was voluntary and participants received a $25 gift card as an honorarium for their time.

**Table 1:** Study participants

Simulation and debrief participants (*N* = 13)	Individual, semi-structured interview participants (*N* = 13)
13 faculty obstetricians	12 faculty obstetricians
2 family medicine obstetricians	2 family medicine obstetricians
11 obstetric residents	7 obstetric residents
5 midwives	2 midwives
8 nurses	7 nurses

### Data collection

Data collection took place in three iterative phases. We conducted 13 multi-professional simulation sessions, which included the same acute obstetrical scenario (Additional file 1: Appendix) involving a total of 13 obstetricians (OB), 11 obstetrical residents (OBR), 8 obstetrical nurses (N), 5 midwives (MW), and 2 family medicine consultants (FP). The scenario involved five scripted “challenge moments” (Additional file 1: Appendix) where the OB participant demonstrated specific lapses in judgment (e.g., unwarranted delay in responding to a call for help, requesting a contraindicated medication). The other participants, who were considered low-authority team members in the context of this scenario, were unaware of the OB consultant’s status as a “partial-actor” in the scenario. We deliberately asked the OB consultant participants to imagine enacting these lapses during a particularly stressful or overwhelming call shift and to avoid overt rudeness or other disruptive behavior (Additional file 1: Appendix). All participants took part in a structured debrief after the scenario, following the PEARLs debriefing framework [12], which was recorded and transcribed verbatim. All debriefs were led by TT. The deception was disclosed at this point. The deception was a necessary aspect of study design to enable our OB consultant participants to authentically experience team dynamics during scripted challenge moments. We followed best practices before, during, and after the simulation to do what we could to preserve psychological safety given the necessary deception. Team members observing the simulation and debriefing completed field notes to capture their initial impressions and insights.

After completing the simulation scenario and multi-professional debrief, all participants were invited to participate in a virtual follow-up, in-depth, one-on-one interviews. Interviews were conducted by TT, RP, and LC. RP conducted the majority of the interviews with low-status team members as she does not have a pre-existing professional or training relationship with any participants. In total, 30 follow-up interviews were conducted, including 12 OB, 7 OBR, 7 N, 2 MW, and 2 FP. Following our simulated primed inquiry approach, participants were shown clips from the simulation (i.e., the moments around a particular error) and were asked to reflect on the experience. Participants were also asked open-ended questions regarding their perceptions and experiences with challenging or being challenged by other team members both in the simulation and their clinical practice, particularly when there was a perceived power differential. Interviews were between 45 and 60 min in length. Interviews were audio-recorded with the participant’s consent and transcribed verbatim.

The study received ethical approval from the local institutional review board [REB #115445]. Informed consent was obtained from all participants and participants were assured of their confidentiality and the voluntary nature of their participation. All data were stored securely, and transcripts were de-identified to protect the privacy of the participants.

### Data analysis

Data analysis was conducted in accordance with Braun and Clarke’s six-phase approach to inductive thematic analysis [13]. TT and RP reviewed all transcripts from the debriefs and individual interviews throughout the iterative process to familiarize themselves with the data set and developed an initial set of codes using Nvivo software. Following a series of analytic conversations, our analysis moved from the descriptive to the conceptual as we identified, developed, and refined themes. Our data collection and analysis ceased once we determined that our themes had clear boundaries and sufficient conceptual depth [14] and their explanatory power was confirmed through researcher crystallization [15].

### Reflexivity

In keeping with our constructivist, inductive approach our findings are co-constructed and a product of interplay between the data and our unique perspectives as researchers. Our research team is composed of two obstetricians (TT, HB), a sociologist (RP), a midwife (LC), a pediatric surgeon (NS), and a doctoral student (THD). We have a shared interest in interprofessional team dynamics and communication. For the clinicians in our team, this work is informed by lived experiences as healthcare providers and team leaders within interprofessional teams, while other team members approached this from the patient’s perspective. We have worked to leverage the diversity of our perspectives throughout the research process.

## Results

Across the 13 simulation scenarios (see Table 1), we noticed very few direct challenges. Instead, most of the communication we observed around the challenge moments was direct, subtle, and sometimes non-verbal. For the purposes of our analysis, we defined direct challenges as explicit expressions of concern, using the advocacy inquiry framework described by Pian-smith et al. [3], and indirect communication as all other forms of communication (both verbal and non-verbal) in response to a challenging moment.

As reported in our previous work [16], the lack of direct challenges was initially both surprising and unsettling for team leaders. Given the obvious patient safety threats associated with the five “challenge moments,” team leaders expected to receive advocacy inquiry challenges that were formulated with clear and direct language. Instead, low-authority team members either accepted the team leaders’ errors or the communication observed was subtle, indirect, and often oblique. During the debriefs, team leaders came to understand that team members’ indirect communication around the challenge moments was intended as challenges. Through reflection and exploration in the debrief and the subsequent follow-up interviews, team leaders came to recognize these indirect challenges as desirable ways for team members to draw their attention to potential errors or issues and subtly say “what are you doing about this” (OB6). Team leaders and team members ultimately indicated that they preferred indirect challenges in practice, in part because of the presence of an awake patient and the desire to appear as a competent, unified team. Furthermore, we identified various assumptions about competent teams that precluded direct communication strategies. Using verbatim quotations from the team debriefing and individual interview transcripts, we will elaborate on these findings using de-identified participant codes (OB###-obsetrician, OBR###-resident, MW###-midwife, N###-nurse, FP###-family medicine consultant).

### Direct vs. indirect challenge scripts

Our analysis highlighted the pervasive expectation that team members would not deliver direct challenges in response to poor judgment from the team leader in the patient’s presence. When speaking about explicit or direct challenges, participants described them as “insubordination” (R003), “shocking” (OB011), akin to “confrontation,” and therefore “not quite a safe option” (M010, interview). Explicit challenges were perceived to compromise functional team dynamics, and thus, they were “extraordinarily rare” (OB007, interview). As one OB participant explained:

The direct thing can go badly for people…unlike general surgery where the patient is asleep, all of this communication is happening in front of an awake patient with a family member present. So, it can be hard... you’re always mindful of what’s being picked up on by the other people in the room, including the patient and their partner. (OB004, interview)

### The awake patient

The awake patient was a prominent justification for silence or subtle challenges, even during an acute situation, like the simulation scenario, where professional lapses or errors from a team leader or other team members could dramatically impact the patient’s treatment and potential outcome.

Our analysis revealed how explicit challenges were perceived to undermine deeply held assumptions, as described by our participants, about what patients expect from competent teams. Violation of these assumptions was believed to erode or threaten the patient’s trust in the team. Each of these assumptions is outlined in Table 2.

**Table 2:** Examples of how direct challenges disrupt the image of a competent team

Assumption	Threat	Quotation
Competent teams never disagree or experience awkward moments	Direct challenges threaten interpersonal relationships	“I value the nurses’ challenges, I do. I think that they keep me on my feet… I get the most frustrated and demoralized when they do it in front of the patient… that’s what ruins our interpersonal relationship, the patient’s trust in us, and all of those things that we need to move forward.” OBR003, interview
Competent teams always have things under control	Direct challenges introduce uncertainty	“You want to look like a solid team, even if it’s nursing-anesthesia, whoever the mix of the team is you’re there for one thing. And you don’t want your patient to feel like everybody is against each other or that they’re not working together to care for her…” N005, interview “…and let’s really try not to raise any sense of uncertainty until we know or, if we have an answer and we can actually give them a concrete plan…we all know when you hear a doctor say I don’t know, I’m not sure, that’s really worrisome for the patient.” OB 011, interview
Competent teams do not make mistakes	Direct challenges highlight mistakes	“And you’re trying to navigate between keeping the patient calm and not wanting to make it seem like the person in charge maybe is making not the best choices. And that is a very fine line as a nurse to navigate…” N008, interview “I can’t just turn to my colleague in front of our patient and say you’re wrong. I need to find something that is a little bit more eloquent to not completely sever the trust.” MW010, interview
Competent teams respect hierarchy	Direct challenges disrupt the hierarchy	“Oh, the hierarchy is so deeply built into all of us, that [a direct challenge] would be like swearing at your parents, you just don’t do it.”OBR010, interview “So, you can’t be too loud, you can’t be too vocal, because you don’t want to undermine the patient’s confidence in the team… And so, I tend to not question my consultants in front of patients in order to respect that hierarchy.” OBR007, interview
Competent teams never deviate from the plan	Direct challenges undermine the plan	“But to stop and be like, ‘[OB] I do not agree with you leaving right now,’ as someone is bleeding out, feels a lot of times wrong. It’s just like, okay, well let’s just do the best that we can in this scenario, and we’ll see what happens.”MW002, interview “I can’t see a resident in front of a patient saying, ‘I’m not sure I would give Hemabate.’” OB008, interview

In contrast, indirect challenges were formulated to be palatable and deliberately subtle (Table 3). They were likened to “cues to bring us back, to re-establish that situational awareness…so I think those things do help” (OB007, interview). These approaches were perceived to be so favorable that they had become embedded in the culture of teaching and practice:

I think the good charge nurses will actually train the nurses to use some of those techniques. Just suggesting, ‘hey do you want a PPH [postpartum hemorrhage] kit, or don’t you think maybe we should call NICU, or do you want to move the patient over to the OR now?’ (OB009)

**Table 3:** Typology of indirect challenges

Typology of indirect challenges	Participant example
Expressing personal uncertainty or discomfort	“Could you show me how or could you perform it because I don’t feel comfortable?” OBR006, interview
Reframing challenges as an educational question	“If I think that they’re not doing the right thing, I try and phrase it in like, ‘for my own learning because I don’t know’” OBR009, interview
Providing facts without judgment	“Sometimes … the nurse will say something similar to, ‘the heart rate’s been down at 60 for 3 min now.’ … it gives me a little nudge to say, ‘okay, let’s stop waiting, let’s act.’ Because sometimes when things are happening like that, you get a sense of tunnel vision, and you don’t see the bigger picture. FP008, interview
Non-verbal cues	“Or sometimes you get in the room, and the nurse has already got the O.R. pack stamped up for a C-section, and you’re like, I think I know what you’re thinking.” OB009, interview

Many of our participants were comfortable assuming that the team leader would decode the “gentle prompts” (MW010, interview) and respond accordingly without alerting the patient to the problem at hand. In this example, a nurse participant recounted her approach to the simulation when the team leader was scripted to leave in the middle of a crisis:

You know if you word it like a question then maybe it triggers someone to think, ‘oh yeah, okay, maybe this *is* worse than the labouring patient next door.’ (N012, debrief)

Thus, indirect challenges or “silent reminders” (OB012, debrief) were often framed as a less disruptive, preferable approach to redirecting or signaling disagreement without frightening the patient.

### The risks of indirect challenges

Some participants were uncertain about whether indirect challenges were reliably preferable to more direct or explicit approaches. These discrepant examples often highlighted how indirect approaches could be ineffective if they were ignored or misunderstood, especially if the team leader is “distracted” or “so tired [they’re] not thinking properly” (OB007, interview). One participant recounted a situation where she was caring for a “high anxiety” patient and despite her attempts to convey her concerns to the team without upsetting the patient, the team “wasn’t picking up on the cues of questioning” (N008, interview). In another example, a team member skipped both direct and indirect communication altogether; instead, they decided to consult the OB on-call and completely circumvented the patient’s most responsible provider. The participant justified this lack of communication by explaining:

It’s hard because you don’t want to have an argument in front of the patient and make them question his ability to care for her. So, I feel just by bringing in the other team it might have just seemed a normal thing [for the patient]. (N009, interview)

The few circumstances in which participants could imagine justifying a direct challenge in the presence of an awake patient were high-stakes scenarios where the patient’s life was definitively and immediately at risk.

I don’t think I would ever challenge a person on their management plan of their patient because that’s their patient, unless I thought it was going to be like… like, the patient would die because of it or something. (OBR003, interview)

Outside of these conditions, direct challenges were unthinkable.

And if she wasn’t dying, and it wasn’t an urgent thing, I would really hesitate calling a second staff because… I would feel frowned upon, and then I feel like I still have a lot of residency to go, and then this staff would hate me the whole time… how do you recover from that? (OBR009, debrief)

In this framing, not only were direct challenges seen as risky, but also there were very few situations in which such a significant risk was justified. Considering the perceived interpersonal and professional risks of direct challenges, it is unsurprising that many participants preferred to speak up via indirect or subtle challenges. Participants perceived this mode of communication as a way to reduce the risks associated with speaking up while still raising concerns to their team leader.

## Discussion

This study used an interprofessional simulation scenario to elicit insights from team members about how teams function when a leader is making poor decisions. We found that our low-authority team members were reluctant to explicitly challenge authority, which is a well-established finding in the speaking-up literature [3, 4, 7]. Interpersonal risk can prevent team members from speaking up, even when their personal safety is at stake [17]. Unexpectedly, the predominant reason our participants cited for offering what Bould et al. [2] would classify as a “weak” or indirect challenge, or no challenge at all, was the presence of an awake patient, a finding that remains underexplored in the existing literature. Barlow et al.’s [18] recent work suggests that the presence of others may shape how speaking-up messages are received but the precise nature of this effect is still unknown. Future work should focus on explicating how the presence of others, including awake patients, shapes the delivery and reception of speaking-up messages in contexts beyond obstetrics.

The speaking-up literature has historically dismissed indirect approaches to challenging authority, based on catastrophic and highly publicized cases in which low-authority team members “failed” to speak up explicitly [8, 19]. These tragedies are used to justify ongoing, resource-intensive efforts to train low-authority team members to issue direct challenges—a form of communication that people across many domains consistently struggle to enact [4, 20]. Across a series of simulation-based studies, the ideal speaking-up script within healthcare teams was described as “crisp advocacy/inquiry” which involved expressing an explicit concern along with a question to elicit the team leaders’ mental model [3], informed by the best practices in aviation. While there is certainly merit in empowering low-authority team members to practice these challenge scripts, they are insufficient because they fail to consider the role and influence of team leaders [16] and they overlook a potential role for indirect approaches, particularly when direct approaches are potentially inadvisable, such as in the case of an awake patient.

Our results revealed how, in this study context, the awake pregnant patient was both omnipresent and yet also invisible in the participant’s reflections. The patient was mostly symbolic as an observer or judge and rarely spoken about as a vulnerable entity to be protected at all costs, which raises concern. It is possible that our participants were “discounting the future,” a phenomenon whereby a more certain *immediate* risk (i.e., the threat of disrupting the patient’s perception of the team or team leader’s competence) outweighs the somewhat less certain *future* risk of actual patient harm [20]. This might explain why very few low-authority team members in our study seemed troubled by the notion that maintaining the perception of a competent team was more important than speaking up to correct the team leader’s actions. Previous work by Detert and Edmondson offers another possible lens for making sense of this perplexing finding: implicit voice theories [20]. Implicit voice theories capture deeply held, often subconscious, and sustained beliefs about the impact of voice as it relates to authority. Importantly, such theories need not be accurate to serve a purpose or function. In their initial qualitative field study within a large tech company, Detert and Edmondson [20] elucidated five implicit voice theories. One of them, “Don’t embarrass the boss in public”, is particularly reminiscent of our participants’ motivations [20]. Finally, other studies have revealed that low-authority team members may believe, or want to believe, that they are not medicolegally responsible for poor outcomes if they are following their supervisor’s orders, even if they disagree with those orders [2, 9]. Not only is this fundamentally untrue, but it also places a disproportionate, unrealistic burden on team leaders who are ultimately fallible humans who will inevitably make mistakes.

Until recently, team leaders’ perspectives have been completely overlooked by the existing speaking-up literature [9]. The proliferation of studies focused on teaching low-authority team members to use specific direct challenge scripts all assume that team leaders both want and will respond positively to such challenges [1–4]. Our data suggests this is not necessarily the preferred approach and, in some cases, direct challenge scripts might be received unfavorably by team leaders. This finding aligns with a nascent body of literature dedicated to understanding more about the perspectives of the “receiver” role held by healthcare team leaders that can guide future efforts to train receptive leaders. Receptivity to speaking up messages seems to be influenced by the content of the message and its delivery [9, 21]. Research has found that challenges scripted as non-judgmental statements of facts were received positively by team leaders [9, 21]. Though the authors did not explain what this looked like exactly, it sounds more consistent with what we have described as indirect or subtle challenge language, rather than “crisp advocacy/inquiry” which requires the speaker to express a judgment or concern [3]. More work is needed to elucidate the types of challenge language that routinely circulate among healthcare teams. Critically, team leaders must learn how to tune into the more subtle ways that low-authority team members may be speaking up, particularly while in front of patients or family members.

By using faculty team leaders as partial actors with scripted errors, our simulation design provided an ideal learning opportunity for our faculty. They could see how team members responded, often with nonverbal (i.e., by stepping back) or oblique cues (i.e., “Do we have another option?”) during variably egregious challenge moments. This surprised many faculty—even those who acknowledged the rarity of direct challenges in real life. Most faculty had presumed that the seriousness of the errors would prompt an overt challenge. During the subsequent simulation debrief, a few faculty participants reflected on how these subtle cues could be overlooked, particularly if the team leader was stressed, cognitively overwhelmed, or fatigued; this inspired further discussions about the importance of shared accountability across the team. Training team leaders to be aware of common subtle and indirect challenge scripts and to recognize these challenges as communication events that require a response is an important next step. Additionally, training team leaders to be curious receivers of subtle challenges and to actively solicit the opinions of their team members may be an effective strategy to promote more open and explicit communication.

There is an ongoing role for training and supporting low-authority team members to challenge directly in acute situations. However, psychological safety is a prerequisite for this to be successful. Silence will persist until we have team leaders who are motivated to build and maintain an environment in which it is safe to speak up. In the meantime, at the very least, all team members should be aware of indirect communication strategies that represent oblique challenges from team members. Team leaders should approach such communication strategies with curiosity and view them as an opportunity to engage in conversation about the thought process motivating the challenge. It is clear that both direct and indirect communication are imperfect on their own and are suited to different contexts and situations. Future research is necessary to provide further insight into how and when these approaches can and should be used to ensure optimal impact on team communication and patient care.

## Limitations

Direct and indirect challenges are likely overlapping entities and certainly contextual. We chose not to define these for our participants, but given the predominance in the literature, it was logical to use this lens to inform the analysis. This study was not designed to test or prove whether participants’ simulation performance is a replica of real life, though our participants commonly cited similar real-life situations while reflecting on their behavior during the simulation. Similarly, we did not design a study that would test or assess how team leaders typically respond to challenges. We set out to share the perspectives of participants on how they imagine they would respond in such moments or their recollections of how they have responded in other, similar real-life situations. This work took place in a single institution and will necessarily reflect the local culture of this institution. We anticipate further research which will enable us to explore the transferability of our findings in other contexts.

## Conclusions

Low-authority team members primarily relied on indirect challenge scripts when faced with a problematic team leader, in an effort to preserve the image of a competent team in the presence of an awake patient. During post-event debriefing and follow-up interviews, faculty participants described a preference for indirect challenges from low-authority team members, particularly in front of awake patients. In contrast, in the context of obstetric care, direct challenges were viewed by participants as threatening to patient trust and disruptive to the interprofessional team.

We cannot claim that the indirect challenges our participants preferred in the context of an awake patient are necessarily more effective than the direct challenges featured in existing speaking-up research within healthcare teams. Rather, our findings point to the possibility that all team members may see a role in these indirect approaches and that the “awake patient” factor means that these types of communication warrant further exploration and consideration. While acknowledging the potential risks inherent in indirect approaches, dismissing such communication strategies outright risks missing an opportunity to harness their potential. Training team leaders to be curious receivers of subtle challenges and to actively solicit the opinions of their team members may be an effective strategy to promote more open and explicit communication.

### Supplementary Information

**Additional file 1: Appendix.** Multi-professional simulation sessions

## Data Availability

The qualitative dataset generated during and analyzed during the current study is not publicly available because it contains identifiable participant information. De-identified data and study materials may be made available from the corresponding author upon reasonable request.

## Abbreviations

CM: Challenge moment
OB: Obstetrician
OBR: Obstetrical resident
N: Obstetrical nurse
MW: Midwife
FP: Family medicine consultant
