# Supplementary material for: “The patient is awake and we need to stay calm”: reconsidering indirect communication in the face of medical error and professionalism lapses
Source: Adv Simul (Lond). 2024 May 10;9:17. doi: 10.1186/s41077-024-00293-4 (PMC11084124; doi:10.1186/s41077-024-00293-4)
Supplement: Supplementary file 1 — Additional file 1: Appendix. Multi-professional simulation sessions [file 41077_2024_293_MOESM1_ESM.docx]

APPENDIX

1. Scenario
2. Email to partial-actor participants
3. Table of challenge moments

1.Scenario

Case: Labour dystocia and PPH - VERSION 2.0 (Nursing)

Evaluating hierarchical behaviours in an emergent setting, with the following elements:

Fetal decelerations on oxytocin

Forceps delivery

Postpartum hemorrhage

Authors :Taryn Taylor & Maddison Bibby & Priyanka Singh

Creation date: September 3 2020

Last update: February 2, 2021

Target population: Nursing, Obstetrics

Objectives

| Skill | Objective |
| --- | --- |
| Leadership/ followership | 1. Leadership established and maintained across various phases and locations of the scenario as evidenced by:    1. Leader maintains a global perspective throughout the scenario    2. Leader ensures a shared mental model among team members    3. Leadership transitions (if they occur) are explicit and evident to other team members    4. Leader appropriately listens to feedback to ensure patient safety in complex scenarios 2. Informed by the shared mental model, followers either anticipate what needs to be done (based on prior experience) or request direction from the leader 3. All team members are willing and able to share their perspective (i.e. "challenge" the leader, contribute to "think aloud" problem-solving) |
| Communication | 1. Team employs closed loop communication strategies throughout the scenario 2. Patient handover includes only the pertinent information, conveyed clearly and concisely 3. Major events and critical moments are announced |
| Resource Allocation | 1. As new "responders" arrive on the scene, they either identify and fill an existing gap or seek direction on how they can be helpful (see also: Followership) 2. Leader delegates tasks among team members according to their scope of practice, skills and abilities 3. Leader is open to feedback as it pertains to comfort level with particular tasks allocated |
| Situational Awareness | 1. Any change in maternal or fetal status is identified, acknowledged and responded to (as needed) immediately 2. Discomfort with plans and expected outcomes is recognized and verbalized to the team |

Simulated case summary:

22y G2P0 at 41 weeks 3 days gestational age is being induced in a community hospital as she is post-dates. She is under the care of the obstetrical team. Oxytocin is at 20mU/min and she has progressed to full dilatation. She underwent ARM 24 h ago. She had been pushing for two hours but is now taking a break for 30 mins as she was getting uncomfortable, feeling exhausted and needed an epidural top-up. A new obstetrical team just started their shift. Handover from the overnight team was that baby was OA spines -2 at last check.

Scenario Requirements

| Equipment | | Confederates | Adjuncts  (e.g. Imaging, bloodwork, forms) |
| --- | --- | --- | --- |
| Applied/in use at start of scenario:  **SimMom**  **baby mannequin**  **portable vitals monitor**  **fetal HR monitor**  **epidural catheter and pump**  **Delivery table with sterile delivery instruments**  **IV oxytocin, 20 units/L running at 20 mu/min (18g IV)**  Available (in room)  O2 sat probe  oxygen  urinary catheter (indwelling)  wall suction | Available (outside of room)  PPH Kit: oxytocin, carbetocin, hemabate, misoprostol, ergot  TXA  fluid warmer  Forceps | patient voice | Inpatient chart  Procedure consent form  Epidural record  Admission bloodwork  FHR strip with contractions q2-3 min, uncomplicated variable decelerations with contractions and now complicated variables with most recent two contractions |
|  |  |  |  |

Participants:

| Actual Identity | Role in Scenario |
| --- | --- |
| OBCU Nurse | Self (starts scenario in room) |
| OB Resident | Self |
| Staff OB | Staff on-call (aware of “script” for scenario) |

Time required for simulation:

| Event | Duration |
| --- | --- |
| Set-up | 5 |
| Simulation | 20 |
| Debrief | 30 |

Baseline simulator physiologic state:

| **HR:**95 | **BP:**115/70 | **RR**:12 | **SpO2:**98% |
| --- | --- | --- | --- |
| **Temp:**36.7 | **FHR:** baseline 140  Decels to low 90s with contractions. 2x prolonged decelerations after contractions. | **Other:** | |

| **Neuro** | Alert + oriented |
| --- | --- |
| **Resp** | GAEB |
| **CVS** | Normal sinus rhythm, S1S2, no murmurs |
| **GI** | N/A |
| **GU** | Foley catheter draining clear urine |
| **Other** | Patient in lithotomy position and pushing with contractions, epidural in situ with pump on IV pole next to patient; oxytocin running at 20mu/min |

Full background information for scenario:

(For the information of instructors/confederates only. Information provided to participants as appropriate for scenario)

| **HPI** | Diane Pipers is a 22y GTPAL 2-0010 who has presented for IOL due to post-dates at 41wks 3 days with successful ARM (24h ago) by the OB team the prior evening and now on oxytocin at 20 mu/min. During the last check the chief OB resident stated the patient was fully dilatated, OA, spines -2 which was about 2 hours prior. Diane is taking a break for 30 mins while her epidural top-up kicks in. The pregnancy has been healthy and uneventful. GBS –ive, RH pos. | |
| --- | --- | --- |
| **OB History** | Spontaneous abortion at 9wks GA 2 years prior, required D&C. Pregnancy conceived via IVF. | |
| **PMHx** | Asthma (no prior hospitalizations), uses inhalers intermittently | |
| **ROS** | Becoming exhausted with pushing and was feeling sharp pelvic pains, otherwise non-contributory | |
| **Meds** | PNV, ventolin PRN | |
| **Allergies** | NKDA | |
| **P/E** | **General** |  |
|  | **Wt/Ht** | BMI 30 |
|  | **Vitals** | HR 90, BP 115/70, RR 12, SaO2 98%, Temp 36.7 |
|  | **CNS/LOC** | Alert + oriented |
|  | **CVS** | Regular strong pulses bilaterally, normal HS |
|  | **Resp/Airway** | No concerns, MP2, normal MO, CROM, full teeth |
|  | **Abdo** | Non-tender, strong palpable contractions |
|  | **Pelvic** | Fetal head is OP, at spines |
| **Investigations** | Admission bloodwork: Hb 100, WBC 14, Plt 145 | |

Information for participants:

Instructions about what information should be given to participants, including background and instructions regarding behaviour, scripted phrases, trigger points, etc.

| **Participant** | **Initial information provided** |
| --- | --- |
| **OBCU Nurse (in room)** | You have just started your shift and you’ve received handover from the overnight nurse who has been caring for her  Course in labour: Admitted 24 h ago at 3cm dilatation and ARM was completed for clear fluid. She received epidural shortly after. She did not progress past 5cm on her own so oxytocin was initiated and gradually titrated to 20mU/min. She reached full dilatation about 2 hours ago. She pushed for two hours but became exhausted with sharp pains felt despite her epidural. The night OB team offered her a rest while an epidural top-up was administered. On your most recent assessment, her contractions were still strong to palpation |
| **OB resident** | You are on call in a community hospital just starting your shift for the day. |
| **OB staff** | You are the obstetrician on call in a busy community (level II) hospital . You are aware that there there is patient on the board who has been slow to progress.  When you are called to assess the patient, as part of the scenario, we would like you to do the following:   - **When called by OB resident, your initial response will be that you will come when you’re free, purposely ambiguous.** - **Incorrectly assess the baby’s position to be OA** (it will actually be OP) - **Decide to perform a forceps assisted vaginal delivery without conducting patient consent first and without emptying the bladder or calling for peds resuscitation team** (you can act as though the urgency of the situation precludes these steps) - **Only provide assistance to the OB resident with the forceps delivery if directly requested** - **There will be a PPH following delivery. Request hemabate** (you don’t know that the patient has a history of asthma) - **After the hemabate is administered, remove yourself from the room as the hemorrhage is ongoing and intensifying**, stating to the team “I’m going to check on room 220, looks like you’ve got this under control” |

　Scenario timeline:

Stage 1: Initial response to and management of atypical fetal heart rate tracing

| **Objectives addressed by this stage:** (1) Clear concise handover to responders (2) situational awareness indicated by initiating resuscitative measures for fetus (3) Appropriate delegation of tasks among the team when severity of situation recognized | | | |
| --- | --- | --- | --- |
| **Key event(s) in this stage**: (1) Identification of abnormal fetal heart rate tracing on CEFM (2) Seek help from OB team (3) Initiate initial resuscitative measures | | | |
| **Patient information** | **Scenario adjuncts**  **(e.g. confederate tasks, environmental cues, results)** | **Expected Behaviours**  **(i.e. observed and ‘what if’)** | **Progression if expected behaviours not met** |
| History/condition:  Patient awake/alert, becoming anxious, exhausted, epidural is working more effectively since the top-up | CEFM showing complicated variable decelerations when nurse starts in the room.  Prolonged decelerations x2 when in room. If pushing, prolonged decelerations following. | **Nurse –**  Requests help from OB when atypical tracing recognized and becoming abnormal  Considers initial resuscitative measures:   - Oxygen mask to mom if hypoxic - Fluid bolus if hypovolemic - Change position - +/- cervical check for position (may or may not note that position is OP, rather than OA)   Provides focused/succinct handover to incoming resident including what assistance is needed | When initial call for help is received, charge nurse will inform caller that OB is just finishing a delivery but a resident is on their way to assess. There are no nurses currently available to provide a second set of hands |
| Vital signs:  HR 100, BP 110/60, RR10, 98% RA and T37.2 |  | **OB resident**  Ensures initial intrauterine resuscitation steps have been taken (incl. Stopping oxytocin if not yet done)  Conducts a focused history  Re-checks position of fetal head, discovers that it is OP, and attempts to get patient to push (or may attempt a manual rotation)  Calls for help from OB staff to expedite delivery urgently due to abnormal FHR tracing  Provides succinct and complete handover to staff including prolonged labour, hx of asthma and OP positioning | OB staff initial response will be to ignore the urgency of the situation (e.g. “I’ll be by when I’m free”)  If resident accepts that and doesn’t challenge further, the FHR tracing will worsen - prolonged, frequent decelerations. |
| Physical Exam:  Unchanged from baseline, strong palpable contractions. Fetal head OP at spines +1. |  |  |  |
| Other:  Mom states she is still exhausted and sore, cannot provide a lot of maternal effort with pushes. |  | **OB Staff**  Will receive handover from OB resident and will reply that they will come “when free” (which is Stage 2) |  |

Stage 2: Response to and management of forceps application, noted lack of procedural consent

| **Objectives addressed by this stage:** (1) Clear handover to OB staff of cervical exam findings and abnormal heart rate tracing (2) Recognition of medicolegal concern with no procedural consent (3) Proper management and initiation of forceps application and delivery | | | |  |
| --- | --- | --- | --- | --- |
| **Key event(s) in this stage**: (1) Identification of need for forceps delivery (2) Seek help from OB (3) Initiate initial resuscitative measures | | | |  |
| **Patient information** | **Scenario adjuncts**  **(e.g. confederate tasks, environmental cues, results)** | **Expected Behaviours**  **(i.e. observed and ‘what if’)** | **Progression if expected behaviours not met** |  |
| History/condition:  Patient still awake/alert but becoming increasingly more anxious. | CEFM continues to show prolonged decelerations after pushing/contractions and rising baseline FHR when not pushing to 160s.  Labour/pushing progresses but head not descending well.  Forceps tray outside of room.  Successful forceps delivery once properly initiated  Consent form in patient’s chart | **Nurse –**  May offer a shared mental model regarding the continued abnormal FHR tracing.  Offer to help with gathering equipment team may need.  Continue to support the patient as she becomes more anxious. May request consent discussion by team or may provide patient with consent info herself. | Staff will walk in and advise urgent forceps-assisted delivery  Staff will NOT discuss informed consent and immediately ask for the forceps outside the room and start to apply them.  Nurse or OB resident may mention that they are not sure the patient is aware of what is happening/the risks involved OB team and will hopefully advise foley (empty bladder) and peds resuscitation team be notified. |  |
| Vital signs: HR 110, BP 110/70, RR 18, T 38.0 oral and 38.3 axillary. |  | **Obstetrical team –**  Staff will agree to FAVD and initiate gathering the equipment and applying forceps with minimal interaction with patient.  Staff may allow the OB resident to use the forceps themselves for practice, resident will have to ask for help with technique if wanted.  Baby handed off to nurse when born for assessment |  |  |
| Physical Exam:  Pt is exhausted, less power noted with pushes. Becoming warm to touch. |  |  |  |  |
| Other: Infant delivered successfully after forceps application.  Baby crying right away, normal Apgars.  Nurse attends to baby when born – babe does not need any advanced resuscitation |  |  |  |  |
|  |  |  |  |  |

Stage 3: Response to and management of complex postpartum hemorrhage, with staff stepping out of room

| **Objectives addressed by this stage:** (1) situational awareness indicated by initiating resuscitative measures (3) Open communication and positive reception to feedback to ensure patient safety | | | |
| --- | --- | --- | --- |
| **Key event(s) in this stage**: (1) Identification of PPH (2) Realization of PPH severity despite medical management (3) Initiate initial resuscitative measures and medications (4) Staff attempts to leave with assumption of team comfort level | | | |
| **Patient information** | **Scenario adjuncts**  **(e.g. confederate tasks, environmental cues, results)** | **Expected Behaviours**  **(i.e. observed and ‘what if’)** | **Progression if expected behaviours not met** |
| History/condition:  Patient awake, becoming less alert, states feeling “dizzy” | Heavy, unrelenting PVB secondary to atony from labour dystocia despite all medical management applied | **OB resident –**  Assessing amount of blood loss  Considers Ddx PPH, evidence by:  -assessing uterine tone  -exploring uterus for RPOC  -assesses uterus for scar rupture and checks perineum/cervix/vagina for lacerations  -requests bloodwork for coagulopathy  Requests foley catheter | Resident and/or nurse will hopefully remind staff of asthma history and then later express discomfort re: staff deciding to leave and request that they stay for further management as bleeding still not resolved  Staff OB will leave the room if not asked to stay, may be called back |
| Vital signs:  HR 120, BP 80/50, RR16, 96% RA and T38.3 |  | **Nurse—**  Obtains PPH Kit (if not already requested)  Ensures IV Oxytocin flowing well  Offers foley if not yet requested  Administers O2 by face mask and offers to place a second IV  Requests a second set of hands |  |
| Physical Exam:  Fundus 2cm above U, boggy  Airway – MP2, good MO, CROM |  |  |  |
| Other: |  | **OB staff –**  Staff person stays for initial medical management and orders Hemabate (despite asthma hx) as initial uterotonic  Situation not under control but says they are going to leave the room and check in later. “You got this right?” |  |

This scenario will end when decision is made to proceed to the OR for further management (team decides to call in anesthesia from home)

Discussion and Debriefing Guide Template:

| **Error Type** | **Common Errors Observed** | **Teaching Points** |
| --- | --- | --- |
| Crisis Resource Management | **S1:** Failure to involve necessary team members when atypical/abnormal FHR tracing recognized | Importance of intervening if not recovered/resolved. Was a fear of involving more senior members too quickly a concern here? |
|  | **S1:** Did not appropriately involve leaders (senior OB, staff OB) in timely manner when tracings not resolving with usual measures | Be aware of your own shortcomings in knowledge, leaders should help when needed for patient safety. Is there a reason they were not brought in sooner? |
|  | **S2:** Omit discussion of procedural consent and begins forceps-assisted delivery without recognition of medicolegal concern | Why did the consent not happen? Did intimidation with the staff play a role? Was it the emergent scenario leading the team to forget? |
|  | **S3:** Inability to confront the severity of PPH (eg were the key players all involved, was the severity recognized by the team in a timely manner?) | Should recognize need to intervene with medication administration, and ask for help if the usual measures are not working. Did the staff stating they would leave make you uncomfortable? Did you make that clear? |
|  | ***S4a:** Team does not recognize that the patient is in hypovolemic shock and requires immediate intervention | A team effort required for full situational awareness and appropriate interventions. Who noticed the vitals? Who discussed next steps in management? Was the staff present and why/why not? |
|  | **General Communication:** Was there clear handover to team members throughout the stages in order to relay important information for appropriate interventions?    Were differential diagnoses and decisions about management discussed as a team along the way, respecting input from all members?    Were the patient and partner supported through the process? |  |
| Technical Skills | **S1:** Failure to initiate preliminary resuscitative measures for fetus | Review common initial and subsequent emergent actions that can be taken (ie O2, fluids, position change, d/c oxytocin) |
|  | **S1:** Failure to recognize atypical and abnormal FHR tracings on NST | Review NST tracings with careful attention to what is considered normal/atypical/abnormal |
|  | **S1:** Keeping the oxytocin at 18 | If fetal distress, oxytocin is short-lived drug that can resolve atypical tracings quickly if found to be a culprit. Was this because of staff intimidation? How did it feel? |
|  | **S2:** Improper forceps application and delivery |  |
|  | **S2:** Lack of appropriate team-based discussion about options for delivery of non-descending fetus with distress and decreased maternal effort - eg talking over vacuum vs forceps vs C-section and deciding on best course together | Need to have the knowledge of next steps and appropriate management of unique situations of distress. If you are unsure, should always involve more senior team members |
|  | **S3:** Failure to manually explore uterus or administer medications appropriately (eg route, doses) | Review routes/doses of medications, order to typically try them, reaching out to senior members if unsure |
|  | **S4a:** Does not initiate massive transfusion protocol | Remember trauma management guidelines. Who brought MTP up? Was the team responsive to this feedback? |
| Latent Safety Threats | **S2:** Continue to observe abnormal FHR tracings with no intervention | Should at the very least involve senior team members if unsure of next steps. Why was this not done? |
|  | **S3:** Does not realize the severity of PPH and risk for hypovolemic shock either before or after medications administered | Recognition of shock and listening isn’t to feedback of team and their level of worry is very important for patient safety. |
|  | At any point, allowing the staff to leave or not be present when a clear threat exists and there is a lack of knowledge about further management | What were the main barriers to have the staff involved? How were these combatted? Why is it important to have a staff present in times of distress? |

1. Email to partial-actor participants

*You are the obstetrician on call in a busy community (level II) hospital . You are aware that there is patient on the board who has been slow to progress. You've had a stressful, busy night so far and you're exhausted and a bit irritated. When you are called to assess the patient, as part of the scenario, we would like you to do the following:*

*-* ***When called by OB resident, your initial response will be that you will come when you’re free, purposely ambiguous.*** *(backstory here is that you just finished dealing with a challenging interaction in triage)*

*-* ***Incorrectly assess the baby’s position to be OA (it will actually be OP)*** *(it's a difficult check due to molding/caput.)*

*-* ***Decide to perform a forceps assisted vaginal delivery without conducting patient consent first and without emptying the bladder or calling for peds resuscitation team*** *(you can initially act as though the urgency of the situation precludes these steps)*

*-* ***Only provide assistance to the OB resident with the forceps delivery if directly requested***

*-* ***There will be a PPH following delivery (will occur in part two of the sim in a separate room). Request hemabate*** *(you don’t know that the patient has a history of asthma)*

*-* ***After the hemabate is administered, remove yourself from the room as the hemorrhage is ongoing and intensifying, stating to the team “I’m going to check on room 220, looks like you’ve got this under control”*** *(there’s a patient who’s been fully and pushing for 3 hours)*

**Most importantly, the other participants cannot know that you've been given these directions from us.** In simulation, this is called being a partial confederate. Outside of these specific elements, we want you to respond and act however you normally would. We've provided (what we think are) realistic backstory info to explain why someone might initially, reasonably choose to do/say these things in a real-life situation. If the other team members question or clarify or challenge you, please feel free to respond as you normally would.

Please let me know if you have any questions/concerns at all.

1. Challenge moments

| **Scripted Challenge Moments** |
| --- |
| Delayed response to a call for help |
| Incorrect assessment of fetal position |
| Failure to perform safe preparation for forceps-assisted vaginal delivery |
| Advise to give contraindicated medication |
| Leave team during ongoing critical event |
